# Pathogenic variation in human DNA damage repair genes was originated from the evolutionary process of modern humans

**DOI:** 10.1016/j.gendis.2025.101916

**Published:** 2025-11-01

**Authors:** Jiaheng Li, Bojin Zhao, Zixin Qin, Si Hoi Kou, Jia Sheng Chian, Fengxia Xiao, Huijun Lei, Stephanie Andaluz, Jun He, Siddharth Sinha, Xiaowei Mao, San Ming Wang

**Affiliations:** a Sichuan Key Laboratory of Conservation Biology on Endangered Wildlife, Chengdu Research Base of Giant Panda Breeding, Chengdu, Sichuan 610081, China; b Faculty of Health Sciences, University of Macau, Taipa, Macau SAR 999078, China; c State Key Laboratory of Oncology in South China and Guangdong Provincial Clinical Research Center for Cancer, Sun Yat-sen University Cancer Center, Guangzhou, Guangdong 510060, China; d Center for Biomedical Digital Science, Guangzhou Institutes of Biomedicine and Health, Chinese Academy of Sciences, Guangzhou, Guangdong 510530, China; e Macau Union Hospital, Macau SAR 999078, China; f Department of Cancer Prevention, Zhejiang Cancer Hospital, Hangzhou Institute of Medicine (HIM), Chinese Academy of Sciences, Hangzhou, Zhejiang 310018, China; g College of Food Science and Engineering, Ningbo University, Ningbo, Zhejiang 315832, China; h Institute of Fundamental and Frontier Sciences, University of Electronic Science and Technology of China, Chengdu, Sichuan 611731, China; i Sichuan Provincial Key Laboratory for Human Disease Gene Study and the Center for Medical Genetics, Department of Laboratory Medicine, Sichuan Academy of Medical Sciences and Sichuan Provincial People's Hospital, Chengdu, Sichuan 610072, China

**Keywords:** Ancient DNA, Anthropologic, Cancer predisposition, DNA damage repair genes, Evolutionary origin, Pathogenic variation, Phylogenic

## Abstract

DNA damage repair (DDR) genes play critical roles in maintaining genome stability. However, they are prone to genetic variation, of which pathogenic variation (PV) is a predisposing factor for high risk of cancer development in modern humans. Knowing the origin of DDR PV is critical for understanding the genetic basis and developing strategies against cancer risk for modern humans. So far, there is no consensus on the original sources of DDR PV in modern humans. We performed phylogenic analysis, and the results ruled out non-human species as the original source for the PV in modern humans through evolutionary conservation. We performed anthropological analyses by tracing the PV from modern humans in over 5000 ancient humans spanning the past 40,000 years. We observed a widespread distribution of DDR PV shared between modern and ancient humans. The shared DDR PV was predominantly found in modern non-Africans within the past 10,000 years rather than in modern Africans, highlighting that the arising time should be post the latest Out-of-Africa human migration. We also observed the rich distribution of Portuguese *BRCA* founder PV in Brazilian, highlighting that human admixture facilitated DDR PV transmission globally between ethnic human populations. The shorter arising time of DDR PV was further supported by the haplotyping results of DDR founder PV in multiple DDR genes and the predominant heterozygotic nature of DDR PV. Our comprehensive investigation reveals that DDR PV was mainly originated from the recent evolutionary history of modern humans, and highlights that the high cancer risk caused by DDR PV in modern humans is a by-product of the human evolution process.

## Introduction

### DDR genes and pathogenic variants

The human genome can be damaged by different factors. In order to maintain the integrity of the genome, efficient repair of the damaged DNA is essential. This task is performed by the DNA damage repair (DDR) system formed during the evolutionary process, consisting of nine distinct pathways with hundreds of genes.^1^^,^^2^ These pathways are evolutionarily conserved, exhibiting remarkable specificity in repairing different types of DNA lesions: the base excision repair (BER) pathway targets small DNA lesions; the direct reversal (DR) pathway repairs ubiquitous alkylation damage; the Fanconi anemia (FA) pathway resolves strand cross-link damages; the homologous recombination (HR) and non-homologous end joining (NHEJ) pathways facilitate the repair of double-strand broken DNA; the mismatch repair (MMR) pathway corrects mismatch errors; and the nucleotide excision repair (NER) pathway fixs helix-distorting DNA damage.^1^^,^^2^ The intricate repairing network ensures rapid and efficient DNA repair, thereby maintaining genomic stability and cellular homeostasis.

**Figure 1:**
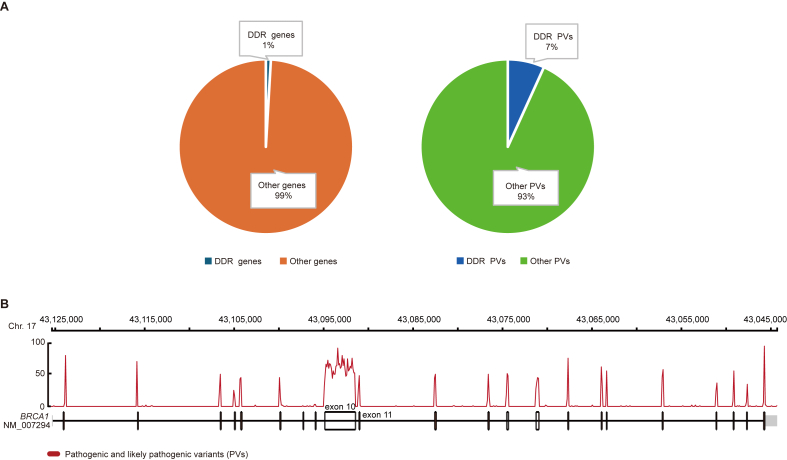
DDR PVs. **(A)** DDR genes and PVs in The ClinVar database. **(B)** Distribution of *BRCA1* PVs in *BRCA1* coding exons. DDR: DNA damage repair; PVs: pathogenic variants.

DDR genes are highly vulnerable to germline variation.^2^ Based on the functional impact of variation, a variant can be classified as Pathogenic, Likely Pathogenic, Variant of Unknown Significance (VUS), Likely Benign, or Benign.^3^ While most variations are benign in promoting survival and fitness or VUS, a portion of the variation is pathogenic (PVs) in damaging the function of the affected DDR genes and related pathways, leading to genome instability triggering pathological consequences. Germline DDR PVs are well-determined as genetic predisposition for elevated risk of human disorders, particularly cancer.^3^, ^4^, ^5^, ^6^ DDR PVs also serve as genetic markers in clinical diagnosis and intervention of cancer.^7^^,^^8^ For example, the PVs in *BRCA* (*BRCA1* and *BRCA2*) damage the function of the homologous recombination pathway in repairing double strand broken DNA,^9^ causing high risk of breast and ovarian cancer^10^, ^11^, ^12^; the PVs in MMR genes (*MLH1, MSH2, MSH6, and PMS2*) impair the function of the DNA mismatch repair pathway, causing high risk of Lynch syndrome, a type of cancer mainly affecting the digestion system.^13^^,^^14^ The PVs in the *BRCA* and *MMR* genes are widely used clinically in the evaluation, intervention, prognosis and prevention of breast, ovarian, and colon cancers.^15^ Since the early 1990s when the correlation between *BRCA1* variation and breast cancer risk was established,^16^ constant efforts have been made to identify DDR PVs and to promote their clinical applications.^17^, ^18^, ^19^ The ClinVar database, one of the major databases in hosting human germline variants including DDR PVs, serves as a reference hub for DDR PV-related clinical applications.^20^ The database encompassed 20,272 pathogenic variants in 91 DDR genes,^21^ including 4,530 PVs in *BRCA1* and 5,648 PVs in *BRCA2* (Fig. 1) (ClinVar database, accessed August 8, 2024).

The note that “nothing in biology makes sense except in the light of evolution” (Dobzhansky, 1973) emphasizes the importance of understanding the nature of biology from evolutionary perspectives.^22^ Knowing the evolutionary origin of DDR PVs is essential for understanding the genetic basis of cancer risk, and developing strategies to control cancer risk in humans.^23^ Despite the germline nature, medical importance, and extensive efforts made in the past decades, however, knowledge for the evolutionary origin of human DDR PVs stays largely unclear. Efforts made so far were restricted by analyzing a small number of variants, mostly benign, in limited DDR genes, and focusing on cross-species conservation through phylogenetic analysis (Table 1). For example, of the 98 *BRCA* variants analyzed in previous studies, only 4 were PVs. As such, the information from previous studies is mostly irrelevant to DDR PVs in modern humans.^24^, ^25^, ^26^, ^27^, ^28^, ^29^, ^30^, ^31^, ^32^ So far, there is no consensus on the evolutionary origin of DDR PVs in humans.

**Table 1:** Previous evolutionary studies on DDR gene variation.^21^

Class (ClinVar)	DDR genes								Total (%)
	BRCA1	BRCA2	XPC	XPA	RAD51	POLD1	LATS1	APC	
Pathogenic	4	1	9	3	1	0	0	4	22 (16.7)
Benign	36	8	0	0	0	0	0	0	44 (33.3)
Uncertain significance	45	2	0	0	0	1	0	0	48 (36.4)
Conflict interpretations	12	2	0	0	1	0	0	0	15 (11.4)
Not provided	1	1	0	0	0	0	1	0	3 (2.3)
Total (%)	98 (74.2)	14 (10.6)	9 (6.8)	3 (2.3)	2 (1.5)	1 (0.8)	1 (0.8)	4 (3.0)	132 (100)

### Evolutionary origin of human DDR PVs

Aiming to fulfill the knowledge gap, we performed a systematic study to investigate the origin of DDR PVs in modern humans.^21^^,^^33^, ^34^, ^35^, ^36^, ^37^, ^38^, ^39^, ^40^, ^41^, ^42^, ^43^ We hypothesized that DDR PVs in modern humans could only originate from three possible sources: 1) From the non-human species through cross-species conservation^44^; 2) From human themselves during the human evolution process^45^; or 3) From both resources.

### The human DDR PV was not from other species

**Figure 2:**
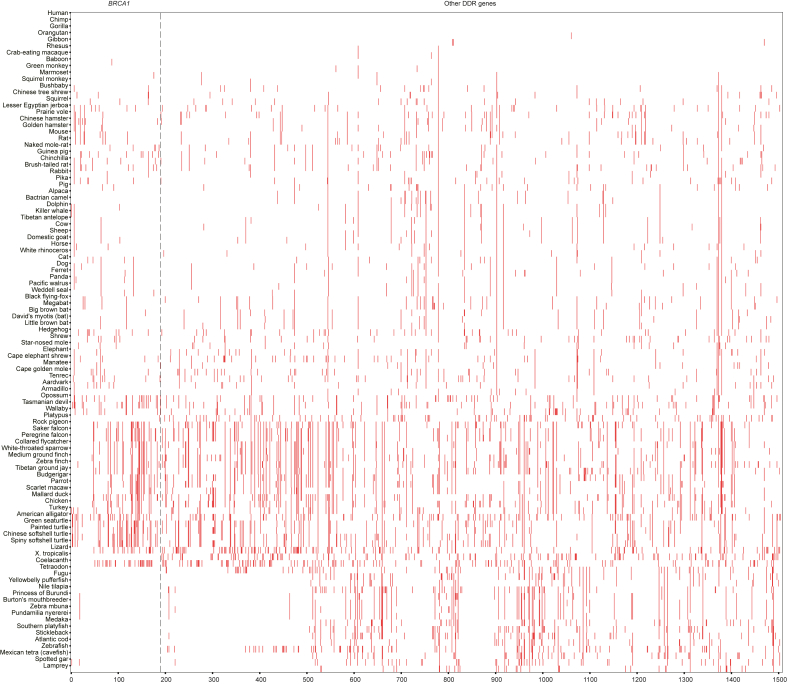
Phylogenetic analysis of DDR PVs. It shows the distribution of the shared 1,504 human DDR PVs in 66 DDR genes in 99 vertebrate species within 8 clades, mostly in the species distal from humans. X-axis: the 1,504 PVs in DDR genes; Y-axis: 99 vertebrate species. Red spots: the human PVs shared with non-human species. DDR, DNA damage repair; PVs, pathogenic variants.

We applied a phylogenic approach to test whether non-human species could contribute to DDR PVs in modern humans. We traced the 7432 DDR PVs identified from modern humans in 99 vertebrate species in eight clades of Fish, Sarcopterygii, Aves, Mammalia, Afrotheria, Laurasiatheria, Euarchontoglires, and Primate. We observed that 1) 79.9% (5,935) of the 7432 PVs were absent in the 99 species. 2) Of the 20.1% (1,497) PVs shared with these species, they were mostly present in the distal species of Sarcopterygii and Aves. For the 53 DDR PVs shared with the species in Primate, 23 (43.4%) were shared with Bushbaby, the species distal to the humans within the Primate clade, and only one PV shared with Orangutan. Gorilla and chimpanzee didn't share any human DDR PVs, although they are the closest species to the humans (Fig. 2). The results demonstrated that DDR PVs in modern humans were unlikely to be inherited from non-human species via cross-species conservation.

### The human DDR PV was originated from human themselves

To test the hypothesis that whether DDR PVs in modern humans could be originated from human themselves, we applied an archeological approach by taking advantage of rich genomic DNA sequences available from ancient humans.

#### Collecting genome sequences from ancient humans

It is well recognized that human genetic variation was largely arisen in recent human history.^46^ However, it mainly refers to the biological adaptation-related, non-pathogenic variation. For the origin of DDR PVs in modern humans, it remains largely unclear. A comparison of genomic sequences between ancient humans and modern humans can provide clues to address the issue. However, it was technically impossible to do so before due to the lack of ancient human genomic sequences. As such, existing studies were mostly restricted to limited founder PVs in a few DDR genes through haplotyping^47^ and molecular clock calculation.^48^ However, the rapid development of ancient DNA extraction technologies in recent years has revolutionized the field. Genome sequences are available now from over 5000 ancient humans dated back to the latest human Out-of-Africa migration 50,000 to 60,000 years before present (BP) and the extinct hominins of Neanderthals and Denisovans.^49^^,^^50^ For the first time, it is possible to trace the origin of modern human genetic variations in ancient humans.^51^^,^^52^ For example, genetic variants in *SLC22A4* predisposing high coronary heart disease risk were identified in a 5300 years old glacier mummy “Iceman Ötzi”,^53^ genetic variants in *EPAS1* enhancing human adaptation to hypoxia were identified in Denisovans,^53^^,^^54^ and genetic traits for human susceptibility to COVID-19 infection and Dupuytren's Disease were identified in Neanderthals.^55^^,^^56^ However, the studies have predominantly focused on genetic variations associated with adaptive changes in response to selective pressures from climate, lifestyle, and infectious diseases etc. No attempts have been made to address the origin of DDR PVs that predispose modern humans to cancer, the most devastating threats to human health.

We collected genome sequences from 5031 published ancient human genomes dated from 45,045 to 100 years BP, 12 Neanderthals dated from 120,000 to 38,515 years BP, and 4 Denisovans dated from 158,500 to 69,650 years BP.^21^ We then searched DDR PVs in the ancient humans. The quality of DNA extracted from ancient humans is critical to ensure the reliability of the data generated. Nowadays, the technologies for extracting DNA from ancient humans are highly advanced because of the outstanding work by the leading laboratories and scientists such as Svante Pääbo,^57^ and standard protocols and strict extraction conditions are widely adapted to control the quality of the DNA extracted from ancient humans. The sources of DNA damage in ancient samples have been well determined, such as the exogenous microbe contamination in ancient samples and modern human DNA contamination during the process of DNA extraction; fragmentation of ancient DNA with breaks enriched next to purines; deaminated cytosine (C) to uracil (U) in ancient DNA caused mispairing during PCR amplification, resulting in C to T substitution in the amplified DNA; and oxidized pyrimidines and cross-links blocking amplification of the affected region in ancient DNA. Effective measures have been developed to maximally prevent the influence of these artifacts to ensure the integrity of the extracted DNA and the data derived from ancient DNA. For example, stringent laboratory protocols now cover the entire process of ancient DNA extraction, library preparation, DNA sequencing, and sequence analysis, such as the controlled laboratory environments for DNA extraction, amplification and indexing, and the use of uracil-DNA glycosylase with endonuclease VIII to limit uracil-driven mis-incorporation. Tight quality control during sequence analysis also plays crucial roles in further eliminating the artifacts, such as excluding terminal bases, eliminating duplicates and low-quality sequences, and removing the sequences from contaminated human mitochondria DNA and nuclear DNA. In our studies, we further applied two extra steps to ensure the reliability of DDR PVs derived from the ancient DNA sequences, including the use of binary-based, curated, quality-controlled BAM files rather than the raw text-based FASTQ read files, and the use of well-annotated DDR PVs in the ClinVar database as the references to determine their presence in ancient DNA rather than calling DDR PVs directly from ancient DNA sequences. These extra steps compensated for the inability of performing technical replicate validation due to the limitation of lacking ancient DNA samples.

#### Identifying DDR PVs conserved between modern and ancient humans

We searched the ancient humans for the presence of 1781 DDR PVs in 87 DDR genes identified in modern humans and validated them in ClinVar.^41^ We identified 1266 PVs in 73 DDR genes in 1019 ancient humans (Table 2; Table S1), accounting for 71% (1266/1781) of DDR PVs present in the modern human population. We constructed a database to host DDR PVs from ancient humans (https://genemutation.boboz.io/dbddr-AncientHumans).

**Table 2:** DDR PVs identified in ancient humans.

A. PVs identified in DDR pathways					
DDR pathways	DDR genes	Ancient humans		Modern humans^b^	
		Gene with PVs (%)	No. PVs	Genes with PVs (%)	No. PVs
Fanconi anemia (FA)	49	27 (55)	615	30 (61)	926
Homologous Recombination (HR)	37	21 (57)	636	21 (57)	916
Mismatch Repair (MMR)	22	8 (36)	225	8 (36)	188
Nucleotide Excision Repair (NER)	42	13 (31)	95	13 (31)	163
Nonhomologous end joining (NHEJ)	13	6 (46)	64	7 (54)	129
DNA damage response (DDR)	15	3 (20)	82	5 (33)	86
Base Excision Repair (BER)	32	7 (22)	46	7 (22)	72
DNA replication (DR)	34	12 (35)	36	11 (32)	36
Total^a^	169	73 (43)	1,266	81 (48)	1,781

B. PVs identified in DDR genes			
Gene	PVs	Gene	PVs
*ATM*	147	*RAD51*	5
*BRCA2*	122	*XPA*	5
*BRCA1*	116	*ATR*	4
*FANCA*	67	*DCLRE1C*	4
*MLH1*	66	*ERCC4*	4
*MSH2*	59	*LIG4*	4
*TP53*	50	*NHEJ1*	4
*PALB2*	48	*RAD54L*	4
*MSH6*	46	*RNASEH2C*	4
*RAD50*	38	*SLX4*	4
*MUTYH*	33	*SSBP1*	4
*BRIP1*	33	*XRCC4*	4
*PMS2*	32	*ERCC1*	3
*ERCC6*	30	*FAN1*	3
*BARD1*	28	*RNASEH1*	3
*CHEK2*	28	*TELO2*	3
*NBN*	24	*FANCF*	2
*BLM*	21	*GTF2H5*	2
*FANCC*	17	*LIG1*	2
*RAD51D*	16	*LIG3*	2
*MSH3*	15	*MCM2*	2
*FANCD2*	12	*RBBP8*	2
*RAD51C*	12	*XRCC2*	2
*XPC*	12	*MCM7*	2
*ERCC2*	11	*RAD51B*	1
*FANCI*	11	*DDB2*	1
*FANCG*	10	*DNA2*	1
*MRE11*	10	*FANCL*	1
*ERCC3*	9	*MCM5*	1
*ERCC8*	8	*POLD1*	1
*RNASEH2B*	8	*TOP3A*	1
*ERCC5*	7	*UBE2T*	1
*RNASEH2A*	7	*UIMC1*	1
*FANCE*	6	*UNG*	1
*FANCM*	6	*XRCC1*	1
*NTHL1*	6	*MCM4*	1
*POLH*	5		

DDR:DNA damage repair; PVs:pathogenic variants.

a Nonredundant numbers.

b Qin et al, 2022.^41^

The shared DDR PVs showed the following features:

1. Narrow range of arising time. The majority of ancient PV carriers were dated within the last 10,000 years BP (Fig. 3A) with an enriched range between 5000 and 1000 years BP, except for a few ancient carriers out of the range. The oldest PV was *BRCA1* c.181T > G p.(Cys61Gly) present in a Russia carrier dated 37,470 years BP, and the youngest PVs were *MSH2* c.1204C > T p.(Gln402∗), *MUTYH* c.437G > A p.(Trp146∗) and *RNASEH2A* c.69G > A p.(=) present in a Vanuatu carrier dated 190 years BP (Fig. 3B and Table S1);

**Figure 3:**
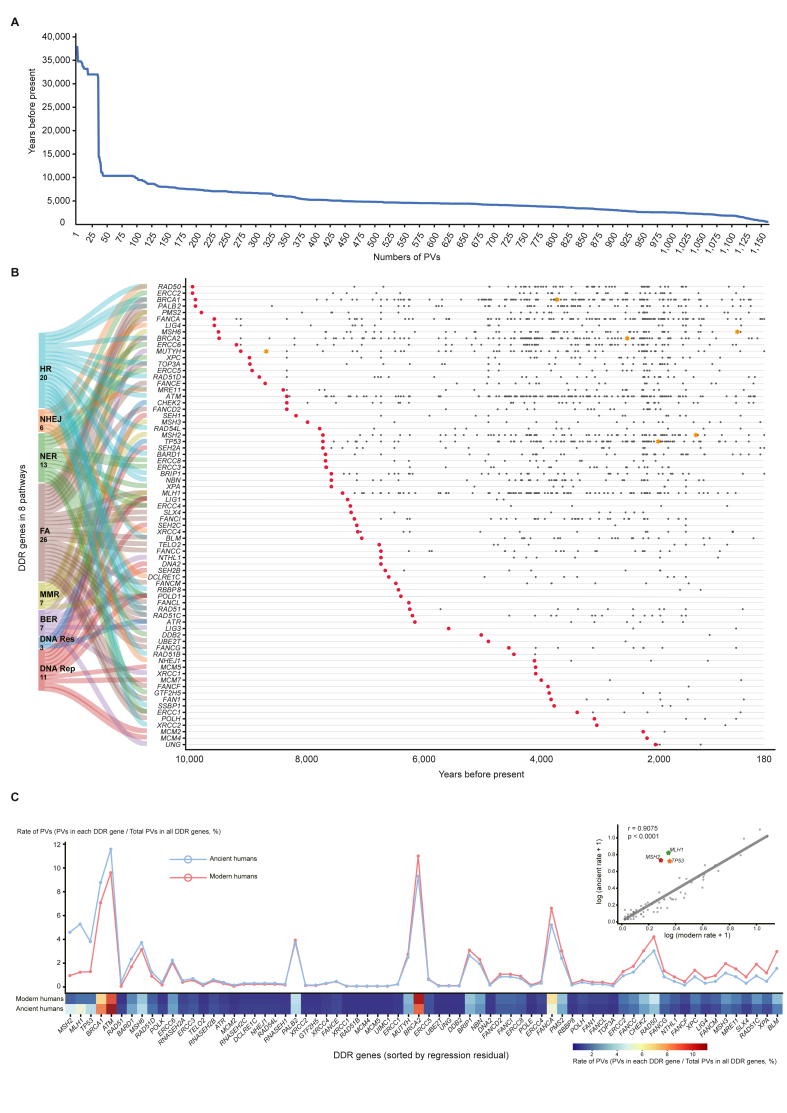
Archeological analysis of human DDR PVs. **(A)** Dated ancient human carriers sharing DDR PVs in modern humans. It shows that most ancient PV carriers were present within the recent 10,000 years BP. X-axis: number of PVs; Y-axis: dated ancient PV carriers (only 1,161 in X-axis, as 110 of the 1,266 ancient samples had no dating information). **(B)** Dated ancient human carriers carrying human DDR PVs in different DDR pathways. It shows the arising time of DDR PVs in different DDR pathways were enriched between 5,000 and 1,000 years BP. Red dot: the first PV identified in a DDR gene; black dot: different PVs after the first PV identified in the same DDR gene; orange star: the earliest founder mutations identified by haplotyping analysis. **(C)** Load of DDR PVs between modern humans and ancient humans. It shows that the loading pattern of DDR PVs was similar between ancient and modern humans, except for the PVs in *MLH1, MSH2* and *TP5*3 that were higher in ancient humans but lower in modern humans. DDR:DNA damage repair; PVs:pathogenic variations.
2. Modern and ancient humans had similar loads of DDR PVs. Comparing with the 1,781 PVs in 81 DDR genes identified in global ethnic populations,^51^ we observed similar quantitative loads of DDR PVs between ancient humans and modern humans, except for the PVs in *TP53*, *MLH1*, and *MSH2*, which showed lower loads in modern humans than in ancient humans (Fig. 3C).

#### Identifying modern human DDR PVs sharing with Neanderthals and Denisovans

**Figure 4:**
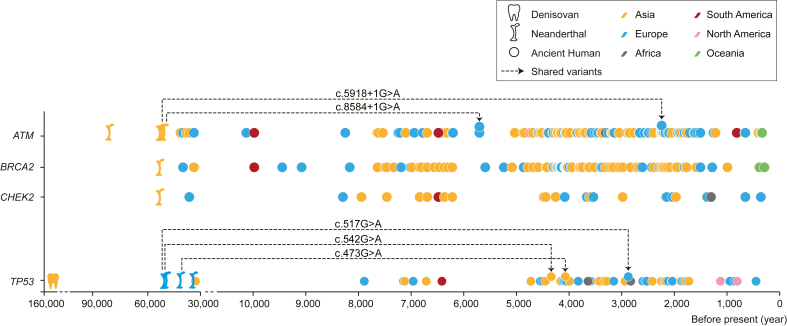
DDR PVs shared between modern humans and Neanderthals. The figure shows DDR PVs in the *ATM*, *BRCA2*, *CHEK2*, and *TP53* genes across different time periods and geographical regions. Dashed arrows indicate specific variants (c.5918+1G > A, c.8584+1G > A, c.517G > A, c.542G > A, and c.473G > A) shared between ancient humans and Neanderthals. X-axis: time ranging from 160,000 to the present (years). Circles with different colors: geographic locations of PV carriers in modern humans. Asia: orange; Europe: blue; Africa: gray; South America: red; North America: pink; Oceania: green. DDR, DNA damage repair; PVs, pathogenic variations.

The human genome inherited 2%–4% of DNA from extinct Neanderthals and Denisovans by admixture.^58^^,^^59^ This suggests a possibility that certain disease susceptibility in modern humans could be originated from the extinct hominins.^60^, ^61^, ^62^ Indeed, we identified a set of modern human DDR PVs in Neanderthals and Denisovans, including 4 *TP53* PVs in three Neanderthals dated 44,000, 42,540 and 38,515 years BP, two *TP53* PVs in a Denisovan dated 158,550 years BP, 3 ATM PVs, 1 *BRCA2* PV and 1 *CHEK2* PV in 5 Neanderthals dated 80,000 to 50,000 years BP (Fig. 4).^21^ These findings suggest that certain cancer predisposition alleles in modern humans were likely originated from Neanderthal and Denisovan.

#### Out of Africa migration was a major landmark for DDR PVs arising in modern humans

Our research supports the emergence of DDR PVs within human populations (Fig. 3A and B). However, the precise time for PV arising remains uncertain considering the long evolutionary history of humans. It is well determined that the genetic diversity of modern humans was strongly influenced by the latest Out-of-Africa migration.^63^ We hypothesized that the Out-of-Africa migration could be a cutting point for the arising of DDR PVs in modern humans due to bottleneck effects, adaptation, and selection after the migration.

**Figure 5:**
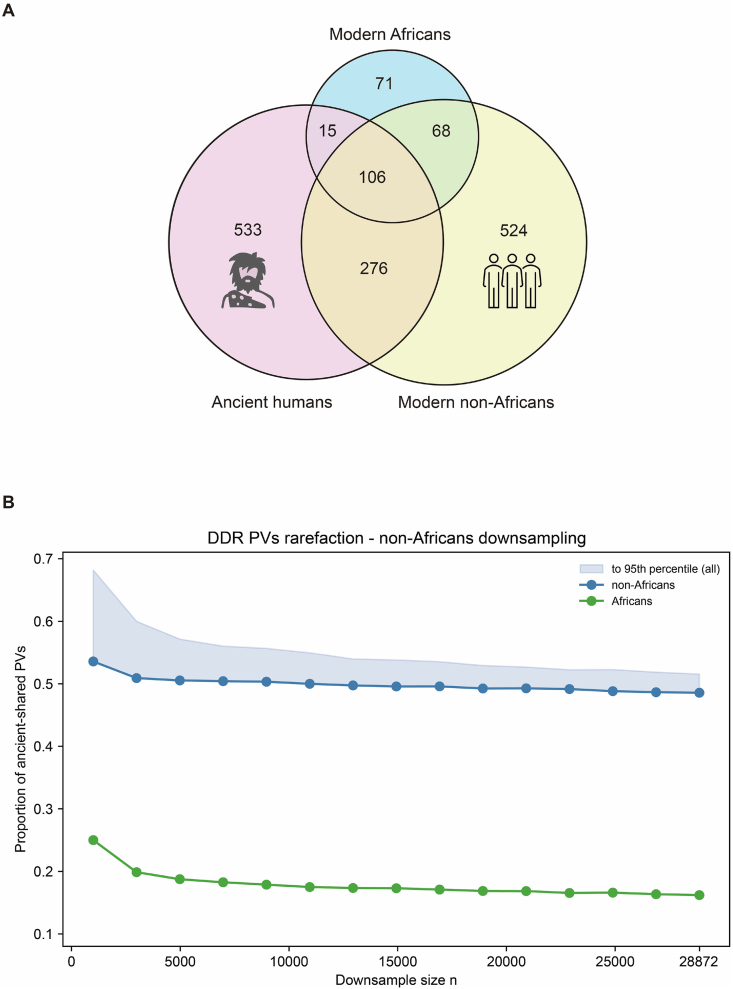
Comparison of DDR PVs among ancient humans, modern Africans, and non-Africans. **(A)** Distribution of DDR pathogenic variants across ancient and modern populations. Venn diagram illustrating the overlap of DDR PVs between ancient humans, modern Africans, and non-Africans. It shows substantially higher variant sharing between ancient humans and non-Africans than between ancient humans and Africans; **(B)** Rarefaction analysis ofDDR PVs among ancient humans, modern Africans, and non-Africans. X-axis: downsized sample numbers. It shows that under the same sample size between non-Africans and Africans, non-Africans shared much more DDR PVs with ancient humans than Africans did. Y-axis: proportion of DDR PVs in non-Africans and Africans present in ancient humans; Circles: the median sharing values across 1,000 samples in each subgroup; lines: DDR PVs shared with ancient DDR PVs following increased sample sizes until 28,872; blue: non-Africans, green: Africans. DDR: DNA damage repair; PVs:pathogenic variants.

To test the hypothesis, we first identified 260 DDR PVs from 28,872 modern Africans and 974 DDR PVs from 222,342 modern non-Africans from public resources. We then used the 1,266 DDR PVs identified from the ancient humans as the intermediator to compare the two datasets. The results show differential sharing of ancient DDR PVs: 70% of ancient DDR PVs were shared with non-Africans only, 26.7% with both Africans and non-Africans, and 3.8% shared with Africans only, suggesting that the majority of DDR PVs in modern humans were closer to the ancient humans than modern Africans did (Fig. 5A). However, the results might be biased due to the different sample sizes between modern Africans (*n* = 28,872) and non-Africans (*n* = 222,342) used in the comparison. To test the possibility, we performed a rarefaction analysis by defining 15 targeted subgroups from *n* = 1,000 to *n* = 28,872 for the 222,342 non-Africans. For each subgroup, we randomly drew *n* individuals from 222,342, and repeated the process 1000 times. We then identified DDR PVs in each subgroup and searched for their presence in ancient humans. The results showed that the ancient-shared PVs in the non-Africans remained remarkedly higher (median = 49.5%, 95th percentile = 51.5%) than those in the Africans (17.3%, 95th percentile = 18.5%) (Fig. 5B), indicating that regardless of balanced or unbalanced sample sizes, DDR PVs in modern humans were closer to the ancient humans than modern Africans did. Therefore, the results support the conclusion that most of DDR PVs in modern humans arose after the Out-of-Africa human migration. Nevertheless, we acknowledge that the limited data from Africans may still influence the accuracy of the conclusion. Future studies incorporating more data from Africans, either ancient or modern, should refine the conclusion more precisely.

#### Human admixture promoted the transmission of DDR PVs between ethnic populations

The global exploration initiated in the15^th^ century by the Europeans caused one of the most extensive admixtures in modern humans.^63^ For example, the global exploration by Portuguese in the 15th century generated more than 20 million admixture population worldwide nowadays with Portuguese heritage, as exemplified by the over 5 million Brazilian population carrying Portuguese heritage.^64^ We hypothesized that disease susceptibility in certain ethnic populations could be transmitted to other populations through admixture. We tested our hypothesis by searching for the Portuguese *BRCA* founder PVs in the Brazilian population. From the 255 *BRCA* PVs identified in 7,711 Brazilians, 29 *BRCA* PVs were found to be originated from the Portuguese, including 14 *BRCA1* Portuguese founder PVs, and 15 *BRCA2* Portuguese founder PVs.^43^ These results confirm that DDR PV-related cancer susceptibility is indeed transmittable through human admixture.

#### Arising timing of DDR PVs validated by haplotyping-determined DDR founder PVs

Our research revealed that human DDR PVs were mainly originated from human themselves in the past few thousand years. Historically, the arising time for many well-known DDR founder PVs has been determined via the haplotyping method.^65^ We used the determined arising time of these DDR founder PVs as the references to validate the arising times of DDR PVs determined by our study. We collected the dated time from 68 founder PVs in seven DDR genes, including *BRCA1, BRCA2, MLH1, MSH2, MSH6, MUTYH* and *TP53*. The arising time for these founder PVs were all within the recent 4,000 years BP except *MUTYH* c.1103G > A dated 8,675 years BP and *MUTYH* c.452A > G dated 7,500 years BP. For example, in the three *BRCA* founder PVs in Ashkenazi Jewish, *BRCA1* 185delAG (c.68_69del) was 1,500–750 years BP,^66^ *BRCA1* 5382insC (c.5266dup) was 1,800 years BP,^67^ and *BRCA2* 6174delT (c.5946del) was 580 years BP^68^; the *BRCA1* c.4035delA in Balts was 1,550 years BP^69^; *BRCA2* c.771_775del in Icelander was 500 years BP^70^; *BRCA2* c.7480C > T and c.8327 T > G in Finnish were 400–200 BP and 220–140 years BP^71^; *TP53* c.1010G > A in Brazilian was 2,000 years BP.^72^ The arising timing determined by haplotyping analysis is well within the scope dated by our anthological analysis, further supporting the short history of DDR PVs in modern humans (Table 3).

**Table 3:** Arose time of DDR founder PVs by haplotyping analysis.^21^

Gene	cDNA	Protein	Pubmed ID	Ethnic population	Years BP
*MUTYH*	c.1103G > A	p.Gly368Asp	23361220	European	8,675
*MUTYH*	c.452A > G	p.Tyr151Cys	23361220	European	7,500
*BRCA1*	c.5503C > T	p.Arg1835∗	35377490	Pakistani	3,800
*BRCA1*	c.3228_3229del	p.Gly1077AlafsTer8	18821011	Italian	3,225
*BRCA1*	c.4096+1G > A	–	37958491	Italian	3,000
*BRCA2*	c.9026_9030del	p.Tyr3009SerfsTer7	12655574	Northeast Spanish	2,760
*BRCA2*	c.156_157insAlu	–	34087993	Portuguese	2,600–2,400
*BRCA1*	c.3331_3334del	p.Gln1111AsnfsTer5	33087180	Iberia	2,400–1,600
*MLH1*	c.306+5G > A	–	20858721	Spanish	2,200
*MUTYH*	c.849+3A > C	–	22865608	Italian	2,075
*BRCA1*	c.4327C > T	p.Arg1443Ter	15883839	Canadian	2,000
*TP53*	c.1010G > A	p.Arg337His	26618902	Brazilian	2,000
*BRCA2*	c.5116_5119del	p.Asn1706LeufsTer5	19949853	Spanish	1904
*BRCA1*	c.5266dup	p.Gln1756ProfsTer74	21119707	European	1,800
*BRCA1*	c.676del	p.Cys226ValfsTer8	26852130	Northeastern Italian	1,720
*BRCA2*	c.3036_3039del	p.Ser1013IlefsTer29	9585613	North American	1,600
*MSH2*	c.1457_1460del	p.Asn486fs	15042510	Southern Chinese	1,560
*BRCA1*	c.4035del	p.Glu1346LysfsTer20	23274591	Baltic	1,550
*BRCA1*	c.3048_3052dup	p.Asn1018MetfsTer8	11781691	Western Swedish	1500
*BRCA1*	c.68_69del	p.Glu23ValfsTer17	26595274	Ashkenazi Jewish	1,500-750
*BRCA1*	c.548-?_4185 + ?del ex9-12del	–	25716084	Mexican	1,440
*BRCA1*	c.815_824dup	p.Thr276Alafs	32025337	Senegal	1,400
*BRCA2*	c.9310_9311del	p.Lys3104ValfsTer6	19949853	Spanish	1,365
*BRCA2*	c.5146_5149del	p.Tyr1716LysfsTer8	19949853	Spanish	1,200
*BRCA1*	c.5309G > T	p.Gly1770Val	35216584	North African	800
*BRCA1*	c.1380dup	p.Phe461IlefsTer19	18215206	Italian	750
*BRCA1*	c.3626del	p.Lys1208_Leu1209insTer	11039575	Finnish	720–460
*BRCA1*	c.1016dup	p.Val340GlyfsTer6	16509964	Norse, Dutch, Italian	600
*BRCA1*	c.1556del	p.Lys519ArgfsTer13	11720839	Norwegian	600
*BRCA2*	c.5946del	p.Ser1982ArgfsTer22	9585613	Ashkenazi Jewish	580
*MSH6*	c.10C > T	p.Gln4Ter	25318681	French Canadian	543
*BRCA1*	c.697_698del	p.Val233AsnfsTer4	11720840	Norwegian	500
*BRCA1*	c.2641G > T	p.Glu881Ter	15146556	South African	500
*BRCA2*	c.771_775del	p.Asn257LysfsTer17	8673089	Icelanders	500
*MLH1*	c.1865T > A	p.Leu622His	20858721	Spanish	425
*BRCA2*	c.7480C > T	p.Arg2494Ter	11039581	Finnish	400–200
*BRCA1*	c.5153-1G > A	p.?	19912264	Spanish	380
*BRCA2*	c.755_758del	p.Asp252ValfsTer24	9585613	North American/France	360
*ATM*	c.6115G > A	p.Glu2039Lys	38017116	Arab	325
*MSH2*	c.-823_1076 + 5984del	–	14871915	North American	313
*BRCA1*	c.5212G > A	p.Gly1738Arg	17902052	Greek	275
*MSH2*	c.2152C > T	p.Gln718Ter	30968502	Portuguese	273
*BRCA2*	c.5771_5774del	p.Ile1924fs	33643918	Southern African	>250
*BRCA2*	c.7934del	p.Arg2645Asnfs	33643918	Southern African	>250
*BRCA2*	c.9118-2A > G	p.?	11039580	Finnish	220–140
*BRCA2*	c.8327T > G	p.Leu2776Ter	11039580	Finnish	220–140
*BRCA1*	c.4096+3A > G	p.?	11039576	Finnish	200
*BRCA1*	c.2685_2686del	p.Pro897LysfsTer5	15010701	Dutch	200
*BRCA1*	c.4097-2A > G	–	11039575	Finnish	<200
*BRCA1*	c.1175_1214del	p.Leu392GlnfsTer5	8571953	Unknown	180

DDR, DNA damage repair; PVs, pathogenic variations.

## Factors contributing to the rapid emergence of human DDR PVs

### Population expansion

The rate of genetic variation in the human genome is rather fixed at 2.5 × 10^−8^ variation per nucleotide site per generation.^73^ It is estimated that there were only about few thousand individuals at the time of the latest human Out of Africa migration 50,000 to 60,000 years BP.^74^^,^^75^ Human population has experienced sustained growth since the Pleistocene–Holocene transition (11,000 years BP), continuing the expansion to its current size of more than 7 billion, due largely to the agricultural revolution initiated eight-thousand years ago^76^ and the industrial revolution initiated several centuries ago.^77^ Under the fixed mutation rate, the great expansion of the human population should largely increase the probability of DDR PV accumulation. Further, the bottleneck effects caused by the small number of Out-of-Africa migrants could also play an important role in enhancing the generation of DDR PVs following population expansion.^78^

### Evolution selection

**Figure 6:**
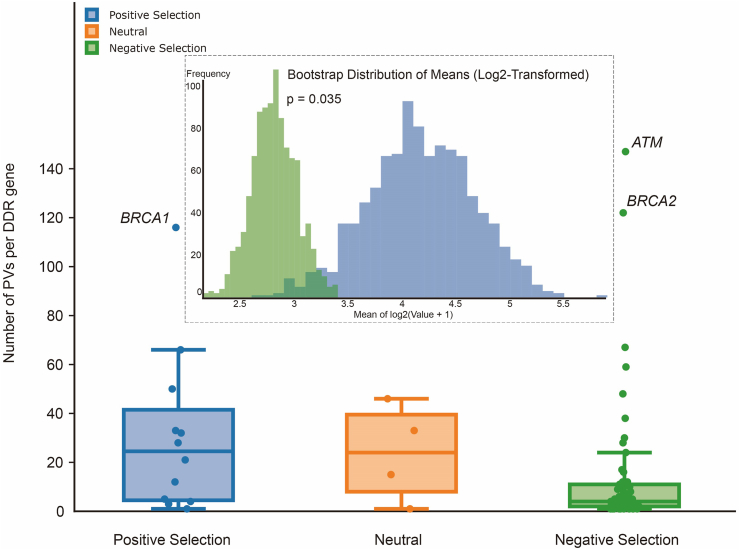
Relationship between different types of evolution selection and the PV quantity in DDR genes. The box-and-whisker diagram shows the number of PVs per DDR gene under positive, neutral, and negative selection. The inset shows the bootstrap distribution of means (log2-transformed) for the positive selection (blue) and negative selection (green) groups (*P* = 0.035). DDR: DNA damage repair; PVs: pathogenic variations.

Evolution selection can also play a significant role in the arising of DDR PVs in the Out-of-Africa migrants and their descendants. DDR genes under positive selection harbor more PVs than those under negative selection (Fig. 6). For instance, human *BRCA1* is under strong positive selection in reflecting its new function gained in regulating immunity,^27^ gene expression and metabolism,^79^ enhancing neural development^80^ and reproduction,^81^ besides its classical function in double-strand DNA break repair. *BRCA1* PVs has high prevalence of 0.2%–0.5% in the human population, or one PV carrier in a few hundred individuals in modern humans.^33^, ^34^, ^35^, ^36^^,^^82^^,^^83^ The positive selection imposed in *BRCA1* may favor *BRCA1* variation, including PVs, for better fitness,^24^ although a trade-off for the PV carriers withhigher cancer risk at their post-reproduction stage.^84^^,^^85^

### Incapable selection due to shorter arisen time

Most of DDR PVs arose over the past few thousand years. This is equivalent to a few hundreds of generations. The shorter arisen time of DDR PVs doesn't allow evolution selection to fully function by either fixing the PVs into the genome or eliminating the PVs from the genome.^86^^,^^87^ This is reflected by the fact that nearly all DDR PV carriers identified in modern humans were heterozygotic.^87^

It is necessary to indicate that the origins of DDR PVs can be very different from those of DDR benign variants (BVs), for which cross-species conservation may exist between humans and non-human species (Table 1).^24^, ^25^, ^26^, ^27^, ^28^, ^29^, ^30^, ^31^, ^32^

Certain limitations exist in our studies. For example, the first PV identified in ancient humans might not be the first PV arisen in ancient humans as older ancient humans were not available in current ancient human samples. Ancient genomic DNA often had lower quality, resulting in false PV calling or missed calling in ancient humans. In addition, certain PVs may not be inherited from ancient human to modern humans but may through spontaneous variation. The limited size of ancient humans may also limit the identification of DDR PVs in ancient humans. The data from Africans are limited that only two ancient Africans of Cameroon were present in the 5031 ancient humans and only 28,872 modern Africans were included in the study, far fewer than the 222,342 modern non-Africans. This could be related to the fact that DNA is hard to be preserved under natural conditions in Africa and the biased human genetics study towards Caucasian populations. More effects are needed to correct the bias for better understanding the nature of human genetics.

## Conclusion

Our systematic study reveals that human DDR PVs are mainly originated from human evolutionary processes, particularly within the past few thousand years. The results highlight that the cancer risk predisposed by DDR PVs in modern humans is a by-product of human evolution.

## CRediT authorship contribution statement

**Jiaheng Li:** Writing – review & editing, Writing – original draft, Funding acquisition, Data curation. **Bojin Zhao:** Writing – review & editing, Data curation. **Zixin Qin:** Writing – review & editing, Data curation. **Si Hoi Kou:** Writing – review & editing, Data curation. **Jia Sheng Chian:** Writing – review & editing, Data curation. **Fengxia Xiao:** Writing – review & editing, Data curation. **Huijun Lei:** Writing – review & editing, Data curation. **Stephanie Andaluz:** Writing – review & editing, Data curation. **Jun He:** Writing – review & editing, Data curation. **Siddharth Sinha:** Data curation, Writing – review & editing. **Xiaowei Mao:** Formal analysis, Funding acquisition, Methodology, Resources, Supervision. **San Ming Wang:** Writing – review & editing, Writing – original draft, Funding acquisition, Data curation, Conceptualization, Supervision, Formal analysis.

## Data availability

Our study used only the published and publicly available sequence data. All the variation data generated in our study are available at https://genemutation.boboz.io/dbddr-AncientHumans/.

## Funding

The study was supported by the grants from the Macau Science and Technology Development Fund (China) (No. 085/2017/A2, 0077/2019/AMJ, 0032/2022/A1), the University of Macau (China) (No. SRG2017–00097-FHS, MYRG2019–00018-FHS, MYRG2020–00094-FHS), the Faculty of Health Sciences, University of Macau (China) (No. FHSIG/SW/0007/2020P, MOE Frontiers Science Center for Precision Oncology pilot grant, and a startup fund) to S.M.W.; Chengdu Research Base of Giant Panda Breeding (China) (No. CPB2025–B15) to J.L.; National Natural Science Foundation of China (No. T2322002), National Science and Technology Major Project of China (No. 2024ZD0523900) and the Sichuan Science and Technology Program (China) (No. 2024NSFTD0032, 2024ZYD0006, 2024NSFTD0032) to X.W.M.

## Conflict of interests

The authors declare no competing interests in the study.

## Appendix A Supplementary data

Supplementary Table 1 List of 1,266 DDR PVs shared between modern humans and ancient humans. Supplementary Table 1
